# Systemic Oxidative Stress Is Increased in Postmenopausal Women and Independently Associates with Homocysteine Levels

**DOI:** 10.3390/ijms21010314

**Published:** 2020-01-02

**Authors:** Arno R. Bourgonje, Amaal Eman Abdulle, Areej M. Al-Rawas, Muna Al-Maqbali, Mohsin Al-Saleh, Marvin B. Enriquez, Sultan Al-Siyabi, Khamis Al-Hashmi, Intisar Al-Lawati, Marian L. C. Bulthuis, Douwe J. Mulder, Sanne J. Gordijn, Harry van Goor, Jumana Saleh

**Affiliations:** 1 Department of Gastroenterology and Hepatology, University Medical Center Groningen, University of Groningen, 9713 GZ Groningen, The Netherlands; a.r.bourgonje@umcg.nl; 2 Division of Vascular Medicine, Department of Internal Medicine, University Medical Center Groningen, University of Groningen, 9713 GZ Groningen, The Netherlands; a.eman.abdulle@umcg.nl (A.E.A.); d.j.mulder@umcg.nl (D.J.M.); 3 Department of Biochemistry, College of Medicine & Health Sciences, Sultan Qaboos University, P.O. Box 35, Al-Khoud 123, Muscat, Oman; areejmohammedrawas@gmail.com (A.M.A.-R.); muna.almaqbali99@gmail.com (M.A.-M.); mmtma149@gmail.com (M.A.-S.); 4 Department of Microbiology and Immunology, College of Medicine & Health Sciences, Sultan Qaboos University, P.O. Box 35, Al-Khoud 123, Muscat, Oman; menriquez@squ.edu.om; 5 Department of Physiology, College of Medicine & Health Sciences, Sultan Qaboos University, P.O. Box 35, Al-Khoud 123, Muscat, Oman; ssiyabi@squ.edu.om (S.A.-S.); kh@squ.edu.om (K.A.-H.); intisarr@squ.edu.om (I.A.-L.); 6 Department of Pathology and Medical Biology, Section Pathology, University Medical Center Groningen, University of Groningen, 9713 GZ Groningen, The Netherlands; m.bulthuis01@umcg.nl; 7 Department of Obstetrics & Gynecology, University Medical Center Groningen, University of Groningen, 9713 GZ Groningen, The Netherlands; s.j.gordijn@umcg.nl

**Keywords:** oxidative stress, postmenopausal women, menopause, free thiols, homocysteine, cardiovascular disease, obesity, risk factors

## Abstract

Oxidative stress plays a pivotal role in the pathogenesis of cardiovascular diseases (CVD). Postmenopausal women have an increased risk of developing CVD due to decreased estrogen availability, which is accompanied by increased oxidative stress. Serum free thiols (R-SH) provide a robust and powerful read-out of systemic oxidative stress. In this study, we aimed to establish serum levels of free thiols and explore associations between free thiols and demographic, clinical, and biochemical parameters related to obesity and the risk for developing CVD in both pre- and postmenopausal women. Serum free thiols were measured in a cohort consisting of healthy pre- (*n* = 223) and postmenopausal (*n* = 118) Omani women. Postmenopausal women had significantly lower levels of serum free thiols as compared to premenopausal women (762.9 ± 85.3 vs. 780 ± 80.9 μM, age-adjusted *p* < 0.001). Women′s age was positively associated with serum free thiol levels in premenopausal women (β = 0.36, *p* = 0.002), whereas an inverse association was observed in postmenopausal women (β = −0.29, *p* = 0.002). Homocysteine levels were significantly inversely associated with serum free thiol levels in both pre- (β = −0.19, *p* = 0.005) and postmenopausal (β = −0.20, *p* = 0.032) women, independent from known cardiovascular risk factors. In this study, we show that postmenopausal women are affected by increased systemic oxidative stress, which independently associates with homocysteine levels.

## 1. Introduction

Oxidative stress is defined as an imbalance between the production of reactive oxygen species (ROS) and the antioxidant capacity, caused by either ROS overproduction or by decreased availability of antioxidant substances [1]. A certain amount of ROS is however needed for a variety of physiological processes in cells such as gene regulation, intermediary metabolism, and mitochondrial function [2,3]. However, excessive production of ROS, overpowering the antioxidant defense system, is known to lead to cellular damage and tissue destruction [4]. Systemic free thiol groups (R-SH, sulfhydryl groups) are thought to play a protective role against oxidative stress through ROS scavenging [5]. Since free thiols are readily oxidized by ROS, systemic oxidative stress can be easily measured as the depletion of serum free thiols [2,6,7]. Thus, high concentrations of serum free thiols are generally representative of a more favorable redox status in vivo.

Although oxidative stress is thought to be involved in several disorders affecting fertility in premenopausal females, it typically increases with age, especially after the menopause [8]. Menopause creates a systemic pro-oxidant state due to decreased production of estrogen, a naturally occurring antioxidant [9,10,11,12,13]. An experimental animal study in mice showed that estrogen protects female mice from developing oxidative stress within the adipose tissue [14]. The beneficial antioxidant effect of high levels of estrogen is believed to be induced by the inhibition of the 8-hydroxylation of guanine DNA bases [9]. Therefore, the current hypothesis is that hormone replacement therapy in postmenopausal females might protect against age-related oxidative stress [15]. In contrast to high levels of estrogen, low levels of estrogen might have a pro-oxidant like effect. However, current studies have not reached a consensus on the exact pro-oxidant role of estrogens [16].

Postmenopausal women are known to have an increased risk of developing cardiovascular disease (CVD) [17]. This could be explained by the loss of the cardio-protective effects of estrogen in menopause [18]. In addition, postmenopausal women are also believed to have higher levels of homocysteine as compared to premenopausal women, which is an established marker of CVD [19]. Sex hormones also play an important role in fat metabolism, thereby strongly influencing body fat distribution. In postmenopausal women, weight gain, subsequently leading to obesity, remains an important health concern, imposing an increased risk of developing CVD. The aim of the current study was to compare systemic levels of oxidative stress, as measured by the serum level of free thiols, in pre- and postmenopausal females. In addition, we investigated the association between serum levels of free thiols and markers associated with the risk of CVD (including homocysteine) in pre- and postmenopausal females.

## 2. Results

### 2.1. Cohort Characteristics

Both groups were compared for their characteristics as presented in Table S1. Mean serum free thiol concentrations were 780 ± 80.9 μM for reproductive women and 762.9 ± 85.3 μM for menopausal women (*p* < 0.001 after correction for age, Figure S1). Concentration ranges of serum free thiols were in accordance with previous studies that used the same detection method [20,21,22,23,24]. Median age of reproductive women was 30 years (interquartile range (IQR) 22–42 years), whereas menopausal women had a median age of 54 years (IQR: 50–59 years). Further characteristics of the study population, divided by tertiles of serum free thiol concentrations, are presented in Table 1 (premenopausal women, *n* = 223) and Table 2 (postmenopausal women, *n* = 118).

### 2.2. Demographics and Anthropometric Measurements

Reproductive women within the lowest tertile of serum free thiol concentrations were of significantly younger age (*p* < 0.001), more often lived in rural areas (*p* < 0.001), were more frequently unmarried (*p* < 0.001), had lower numbers of pregnancies and abortions (*p* < 0.001 and *p* = 0.019), had a lower frequency of gestational diabetes (*p* = 0.002), had a smaller waist circumference (*p* < 0.001), lower waist/hip ratios (*p* < 0.001), and a lower percentage of visceral fat (*p* = 0.002; Table 1). Postmenopausal women within the lowest tertile of serum free thiol concentrations were of significantly older age (*p* = 0.04), had a higher waist circumference (*p* = 0.03), and had a higher percentage of visceral fat (*p* = 0.01; Table 2).

### 2.3. Biochemistry Measurements

Reproductive women within the lowest tertile of serum free thiols had significantly reduced levels of total cholesterol (*p* = 0.04), triglycerides (*p* = 0.02), low-density lipoprotein (LDL; *p* = 0.03), very-low-density lipoprotein (VLDL; *p* = 0.04), and apolipoprotein B (*p* = 0.004; Table 1). No differences were observed in biochemistry measurements between tertiles of serum free thiols for menopausal women. Reproductive women within the lowest tertile of serum free thiols had significantly reduced levels of follicle-stimulating hormone (FSH; *p* = 0.009), whereas testosterone levels (*p* = 0.002) and LH/FSH ratios (*p* = 0.003) were significantly higher. Postmenopausal women did not show differences in hormone levels among tertiles of serum free thiols. Moreover, postmenopausal women within the lowest tertile showed significantly higher levels of homocysteine (*p* = 0.003; Table 2).

### 2.4. Independently Associated Factors with Serum Free Thiol Concentrations

**Figure 1:**
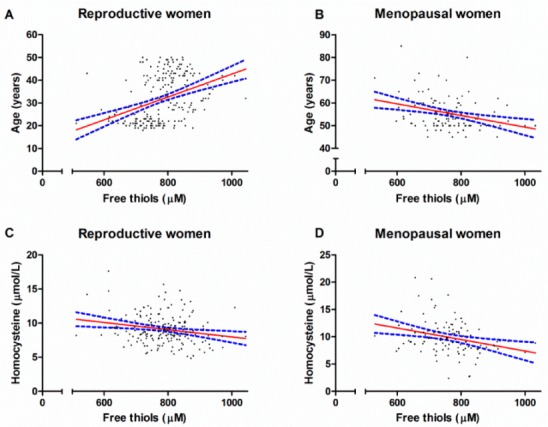
Univariable associations between serum free thiol concentrations and age (**A**,**B**) and serum free thiol concentrations and homocysteine levels (**C**,**D**) among reproductive women and menopausal women.

Univariable and multivariable linear regression analyses were used to identify factors that independently associated with serum free thiols. In the cohort of reproductive women (Table 3), age (β = 0.36, *p* = 0.002), and homocysteine (β = −0.19, *p* = 0.005) independently associated with serum free thiol concentrations. In the group of postmenopausal women (Table 4), the variables age (β = −0.29, *p* = 0.002) and homocysteine (β = −0.20, *p* = 0.03) were independently associated with serum free thiols. Univariable associations between age, homocysteine and serum free thiol concentrations are depicted in Figure 1.

## 3. Discussion

Postmenopausal women have significantly lower levels of serum free thiols as compared to premenopausal women, when adjusted for age. Furthermore, age is positively associated with free thiols in reproductive women and negatively associated with free thiols in postmenopausal women. Moreover, homocysteine was found to be associated with free thiols in both reproductive and menopausal women. This association was found to be independent from other cardiovascular risk factors (i.e., dyslipidemia, glucose, obesity, and estradiol). Homocysteine is an established marker for the risk of cardiovascular events and our results may suggest that serum free thiols are putatively involved in the homocysteine pathway leading to an increased risk of CVD. However, this interesting association should be further investigated in future studies.

The lower levels of free thiols in postmenopausal women are in line with previous studies that have shown that menopause creates a pro-oxidant state in the body due to the decreased production of the antioxidant estrogen [9,10,11,12,13]. The pro-oxidant like effects of estrogen, at low concentrations, include breaks in genetic material, oxidation of bases, and formation of DNA adducts [25]. In addition, serum markers of other oxidants (e.g., malondialdehyde) were also found to be higher in postmenopausal women, as compared to premenopausal women [26]. Moreover, previous studies have shown that oxidized LDL levels are higher and GPx activity is lower in postmenopausal women [27,28]. The current understanding is that the loss of the antioxidant effect of estrogen increases the susceptibility to develop atherosclerosis in postmenopausal women [29]. For instance, previous studies have shown that postmenopausal women have lower levels of nitric oxide (NO) [30], which could subsequently lead to smooth muscle cell proliferation, inflammation, and vasomotor disturbances, thereby increasing the risk of arteriosclerosis [31,32]. In our study, we demonstrated that postmenopausal women had a slightly unfavorable metabolic profile, as compared to premenopausal women. For instance, postmenopausal women in our cohort had higher BMI, higher fat percentages, more unfavorable lipid profiles, and higher HbA1c levels, as compared to premenopausal women. We also found that postmenopausal women had a higher level of homocysteine. These findings are in line with the current postulation that the female menopause can predispose women to an increased risk of developing cardiovascular disease [19,33].

The sulfhydryl-containing amino acid homocysteine was previously established as an important marker of cardiovascular risk [34]. Here, we showed that homocysteine was inversely associated with free thiols in both reproductive and menopausal women. This association was found to be independent of age and other known cardiovascular risk factors (e.g., lipid profile, glucose). Homocysteine is believed to promote the development of cardiovascular disease through several pathways, including proliferation of vascular smooth muscle cells, endothelial dysfunction, oxidative stress, inflammation, and increased synthesis of collagen in the vascular wall [35,36]. Homocysteine is also believed to increase oxidative stress by stimulating the production of ROS (by the upregulation of nicotinamide adenine dinucleotide phosphate) [37], the inhibition of antioxidant enzymes, through the accumulation of asymmetric dimethylarginine (ADMA), and by inhibiting the activity of endothelial nitric oxide synthase (NOS) [38,39,40]. The superoxide anions may react with NO, which leads to the formation of peroxynitrite and a decreased bioavailability of NO [41]. Moreover, endothelial injury may also be induced by the suppression of intracellular glutathione peroxidase activity [42,43]. Collectively, our results might suggest that free thiols are an integrative part of the pathway in which homocysteine can increase the risk of cardiovascular disease.

With ageing, oxidative stress is thought to occur due to the overproduction of reactive species and a decreased ability to neutralize them [44]. With advancing age, mitochondrial dysfunction causes age-related mitochondrial DNA mutations and deletions, subsequently leading to damage. Interestingly, the current study found a positive association between age and free thiols in reproductive women and an inverse association in (post)menopausal women. The inverse association found in (post)menopausal women is in line with previous findings [45,46]. However, the positive association found in reproductive women is rather counter-intuitive. It could be hypothesized that the ageing-induced oxidative stress occurs from a certain age. This hypothesis is supported by our observation that when reproductive subjects <30 years were excluded from the analyses an inverse relationship between age and free thiols (β = −0.21, *p* = 0.03) was present. However, large confirmatory studies investigating the exact age threshold at which oxidative stress starts to play a more prominent role are lacking.

The current study also found that visceral fat was significantly associated with free thiols in postmenopausal women. However, after correction for homocysteine and age, this association lost its significance. A previously conducted study reported that changes in body composition are associated with changes in homocysteine level. Therefore, it could be postulated that the association between visceral fat and free thiols is dependent on the changes in homocysteine level.

Results from the present study are particularly relevant as thiols are amenable to therapeutic modulation, for example, by exogenous administration of thiol-targeted antioxidants [2]. Although these potential interventional strategies have not yet been fully explored, there is evidence suggesting that supplementation with some dietary antioxidants, such as N-acetylcysteine (NAC), is effective in increasing extracellular thiol content by reducing disulphide bonds in proteins [47]. In addition, cysteine supplementation is known to lead to increased remethylation of homocysteine back into methionine at the expense of its catabolism through the transsulfuration pathway [48]. Therefore, dietary intake of sulfur-containing amino acids (SAA) such as cysteine also affects systemic redox status [49]. Moreover, some randomized studies have previously shown that supplementation of antioxidants is associated with a decreased risk of cardiovascular death and risk of myocardial infarction [50]. Although these studies do seem promising, we have to underline the fact that ROS also serve as important signaling molecules for several physiological functions. Given the fact that active lowering of ROS could potentially impact the function of cells, the effects of antioxidant therapy should be carefully monitored.

Our study has several important strengths and limitations. Considering the limitations, this study was of a cross-sectional design, lacking follow-up data or interventions that could have allowed us to more reliably investigate the relationship between serum free thiols and female risk of cardiovascular diseases. In addition, the current study focused on women based in Oman, therefore, the generalizability of our results to other populations is limited. Moreover, the number of premenopausal women included was much greater than the number of postmenopausal women, mainly because it was more difficult to recruit postmenopausal women that fulfilled all study inclusion and exclusion criteria. Furthermore, given the large variation in age between the groups, it remains hard to disentangle the ageing effect from that caused by differences in menopausal state. However, this study was performed on an extensively characterized cohort of pre- and postmenopausal Omani women, including many biochemical and anthropometric measurements.

## 4. Materials and Methods

### 4.1. Study Population

This observational, cross-sectional study was conducted between January 2016 and November 2017. The study included 341 Omani women from Muscat, Sultanate of Oman, who were all recruited via advertisement, which was placed in schools and health centers. Participants were either premenopausal or postmenopausal Omani women aged above 18 years. Premenopause was defined as regular periods and FSH levels ranging between 4.7 and 21.5 IU/mL. Postmenopause was defined according to the following criteria: >45 years old and irregular periods (or halted), or >48 years old and increased FSH levels above 30 IU/mL, or >50 years old and irregular periods. All included women were apparently healthy without any co-morbidity known to affect metabolic profiles of participants. Exclusion criteria were as follows: pregnancy, active or previous smoking, consumption of alcoholic beverages, usage of lipid- or cholesterol lowering drugs, corticosteroids, oral contraceptives, hormone replacement therapy, insulin or any vitamin or hormonal supplements, and the presence of hemoglobinopathies, infectious, or inflammatory disorders. The research ethics committee at the Sultan Qaboos University, Oman, (SQU-EC/164/14, MREC #1019) approved the study and its methodology. All study participants provided written informed consent. The study was conducted in accordance with the principles of the Declaration of Helsinki (2013).

### 4.2. Data Collection

Data were collected through filling out questionnaires containing relevant questions to this study. Demographic and clinical characteristics that were obtained of all participants included age, marital status, place of residence (rural vs. urban), anthropometric measurements including weight (kg), height (cm), body-mass index (BMI, body weight divided by squared height), waist circumference (cm), visceral fat, and fat and muscle percentages. Visceral fat, body fat, and muscle percentages were measured using the Omron Full Body Sensor Body Composition and Monitor Scale, based on bio-electrical impedance [51,52].

### 4.3. Serum Sample Collection, Processing, and Routine Laboratory Measurements

Venous fasting blood samples were collected from all participants. Samples from reproductive women were withdrawn during the first three days of the follicular phase of the menstrual cycle. Samples were put on ice for approximately 1 h directly after withdrawal and then centrifuged for 5 min at 3000× *g*. Serum and plasma were separated from blood cells and stored in 0.4 mL aliquots at −80 °C until analysis. Venous blood samples were collected into two different tubes, a plain tube for measuring hormones, lipid profiles, and fasting glucose, and an EDTA tube for measuring glycated hemoglobin (HbA1c). Routine biochemical parameters were measured in the Clinical Biochemistry lab at Sultan Qaboos University Hospital. Lipid profiles (total cholesterol (TC), triglycerides (TG), low-density lipoprotein (LDL), and high-density lipoprotein (HDL)) and fasting glucose were measured using the Cobas^®^ 6000 Analyzer (c-501 module, Roche Diagnostics, Roche, Basel, Switzerland). Hormone profiles (estradiol, progesterone, follicle-stimulating hormone (FSH), luteinizing hormone (LH), and testosterone) were measured using the Cobas^®^ 6000 Analyzer (e-601 module). Glycated hemoglobin (HbA1c) was measured by an auto-analyzer (Cobas^®^ Integra 400 Plus, Roche Diagnostics, Roche, Basel, Switzerland).

### 4.4. Measurement of Serum Free Thiols

Serum free thiols were measured as previously described, with few modifications [53,54]. Prior to analysis, serum samples were four-fold diluted using 0.1 M Tris buffer (pH 8.2). Subsequently, diluted sample volumes were loaded on a microplate in which background absorption was measured at 412 nm with a reference measurement set at 630 nm using the Varioskan plate reader (Thermo Fisher Scientific, Breda, The Netherlands). After the addition of 20 μL 1.9 mM 5,5′-dithio-bis(2-nitrobenzoic acid) (DTNB, Ellman’s Reagent, CAS-number 69-78-3, Sigma-Aldrich Corporation, St. Louis, MO, USA) in phosphate buffer (0.1 M, pH 7.0), plates were incubated for 20 min at room temperature. Then, free thiol groups were measured by a second sample absorbance measurement. Final concentrations were determined by parallel measurement of an L-cysteine calibration curve (CAS number 52-90-4, Fluka Biochemika, Buchs, Switzerland) with a concentration range of 15.6 to 1000 μM in 0.1 M Tris/10 mM EDTA (pH 8.2).

### 4.5. Statistical Analysis

Data were analyzed using SPSS Statistics 25.0 software package (SPSS Inc., Chicago, IL, USA) and visualized using GraphPad Prism version 5.0 (GraphPad software, San Diego, CA, USA). Demographic and clinical characteristics of the study population were presented as mean ± standard deviation (SD), median (interquartile range (IQR)), or proportions *n* with corresponding percentages (%). Normality testing was performed by plotting histograms and normal probability plots (Q–Q plots). Continuous variables were compared between groups using independent sample *t*-tests and one-way analysis of variance (ANOVA) or Mann–Whitney *U*-tests and Kruskal–Wallis tests in case of non-normally distributed variables. Nominal variables were compared between groups using chi-square tests or Fisher′s exact tests, as appropriate. Study participants were divided into two groups according to the above stated definitions: (1) reproductive women and (2) menopausal women. For each cohort, study participants were divided into tertiles of serum free thiol concentrations and were compared for demographic, clinical, and biochemical characteristics. Univariable and multivariable linear regression analyses were conducted to identify parameters that were independently associated with serum free thiol concentrations. Variables that were statistically significant in the univariable analyses were further analyzed in the multivariable linear regression analyses (enter method). The obesity markers (i.e., BMI, waist circumference, waist/hip ratio, fat percentage, and visceral fat) can cause problems of co-linearity if included together; therefore, these variables were included separately into the model. Two-tailed *p*-values ≤ 0.05 were considered as statistically significant.

## 5. Conclusions

In conclusion, this study showed that postmenopausal women had a significantly lower level of free thiols as compared to premenopausal women, when adjusted for age. In addition, we demonstrated that homocysteine was independently associated with free thiols in both reproductive and menopausal women. Our findings further confirmed that oxidative stress was associated with the risk of developing cardiovascular disease. Future studies are warranted to further investigate the specific pathway in which free thiol balance and homocysteine could lead to a higher cardiovascular risk profile.

## Figures and Tables

**Table 1:** Cohort characteristics of reproductive women (*n* = 223) with comparison between tertiles of serum free thiol levels.

Variables	1st Tertile	2nd Tertile	3rd Tertile	*p*-Value ^†^
	<746.8 μM	746.8−813.9 μM	>813.9 μM	
	*n* = 74	*n* = 74	*n* = 75	
Free thiols (μM)	693.7 ± 48.3	781.0 ± 19.6	865.2 ± 46.3	<0.001
Age (years) *	22.5 [21.0; 27.0]	33.0 [21.8; 42.0]	38.0 [29.0; 43.0]	<0.001
Place of living				<0.001
Rural	33 (44.6)	19 (25.7)	7 (9.3)	
Urban	41 (55.4)	55 (74.3)	68 (90.7)	
Marital status				<0.001
Unmarried	52 (71.2)	26 (35.1)	14 (18.7)	
Married	20 (27.4)	45 (60.8)	60 (80.0)	
Widow	0 (0.0)	2 (2.7)	0 (0.0)	
Divorcee	1 (1.4)	1 (1.4)	1 (1.3)	
No. of pregnancies *	0 [0; 0]	2 [0; 4]	4 [1; 6]	<0.001
No. of abortions *	0 [0; 0]	0 [0; 0]	0 [0; 0]	0.019
Gestational diabetes				0.002
Yes	2 (2.7)	8 (10.8)	16 (21.3)	
No	72 (97.3)	66 (89.2)	59 (78.7)	
BMI (kg/m^2^)	25.1 ± 5.3	26.1 ± 6.0	27.2 ± 6.3	0.110
Waist circumference (cm)	76.1 ± 15.6	85.3 ± 18.0	88.7 ± 18.7	<0.001
Waist/hip ratio *	0.77 [0.72; 0.86]	0.90 [0.82; 0.96]	0.88 [0.82; 0.93]	<0.001
Fat percentage *	38.1 [32.2; 45.2]	40.6 [30.9; 47.7]	42.3 [34.9; 47.5]	0.127
Muscle percentage *	25.2 [22.8; 26.5]	24.3 [22.4; 26.7]	23.4 [22.0; 25.0]	0.059
Visceral fat *	5.0 [3.0; 7.0]	6.0 [3.0; 9.0]	7.0 [4.0; 9.0]	0.002
Biochemistry				
Total cholesterol (mmol/L)	4.6 ± 1.1	4.8 ± 0.9	5.1 ± 1.2	0.040
Triglycerides (mmol/L) *	0.7 [0.6; 1.0]	0.9 [0.7; 1.1]	0.8 [0.7; 1.2]	0.018
LDL (mmol/L)	2.8 ± 1.0	3.0 ± 0.7	3.2 ± 1.1	0.027
HDL (mmol/L) *	1.4 [1.3; 1.6]	1.3 [1.2; 1.6]	1.3 [1.1; 1.6]	0.143
VLDL (mmol/L) *	0.3 [0.3; 0.5]	0.4 [0.3; 0.5]	0.4 [0.3; 0.6]	0.036
Glucose (mmol/L) *	5.5 [5.2; 5.9]	5.6 [5.2; 5.9]	5.7 [5.2; 6.0]	0.953
Apo A1 (g/L) *	1.6 [1.4; 1.8]	1.5 [1.4; 1.7]	1.6 [1.4; 1.8]	0.522
Apo B (g/L) *	0.8 [0.7; 1.0]	0.9 [0.7; 1.0]	0.9 [0.8; 1.1]	0.004
Homocysteine (μmol/L) *	9.1 [8.1; 11.0]	9.0 [7.8; 10.0]	8.4 [7.4; 10.0]	0.140
HbA1c (%) *	5.3 [5.1; 5.6]	5.4 [5.1; 5.6]	5.3 [5.1; 5.6]	0.930
Insulin resistance (HOMA-IR) *	2.3 [1.7; 3.0]	2.7 [1.7; 3.9]	2.1 [1.4; 3.1]	0.178
Hormone levels				
FSH (mIU/mL) *	6.2 [5.0; 7.2]	6.4 [5.5; 7.8]	7.3 [5.9; 9.3]	0.009
Estradiol (pg/mL) *	36.6 [28.8; 48.3]	39.4 [29.5; 48.8]	36.6 [23.2; 45.8]	0.454
Progesterone (ng/mL) *	0.6 [0.4; 0.7]	0.5 [0.3; 0.7]	0.4 [0.3; 0.6]	0.054
Testosterone (ng/mL) *	0.26 [0.18; 0.40]	0.23 [0.15; 0.34]	0.21 [0.11; 0.27]	0.002
LH/FSH *	1.0 [0.7; 1.3]	0.9 [0.7; 1.3]	0.7 [0.6; 1.0]	0.003

Data are presented as mean ± standard deviation (SD) or proportions *n* with corresponding percentages (%). * Skewed variables are presented as median [interquartile range]. ^†^ *p*-values were two-tailed and calculated using one-way ANOVA or Kruskal–Wallis tests, as appropriate. *p*-values < 0.05 were considered statistically significant. Abbreviations: BMI, body mass index; LDL, low-density lipoprotein; HDL, high-density lipoprotein; VLDL, very-low-density lipoprotein; HbA1c, hemoglobin A1c; FSH, follicular stimulating hormone; LH, luteinizing hormone.

**Table 2:** Cohort characteristics of menopausal women (*n* = 118) with comparison between tertiles of serum free thiol levels.

Variables	1st Tertile	2nd Tertile	3rd Tertile	*p*-Value ^†^
	<746.7 μM	746.7−815.6 μM	>814.6 μM	
	*n* = 39	*n* = 40	*n* = 39	
Free thiols (μM)	673.0 ± 45.5	762.7 ± 19.5	853.2 ± 57.1	<0.001
Age (years) *	58.0 [51.0; 62.0]	54.0 [50.3; 56.8]	53.0 [50.0; 56.0]	0.043
Place of living				0.064
Rural	4 (10.3)	1 (2.5)	0 (0.0)	
Urban	35 (89.7)	39 (97.5)	39 (100.0)	
Marital status				0.775
Unmarried	0 (0.0)	0 (0.0)	0 (0.0)	
Married	32 (82.1)	36 (90.0)	32 (82.1)	
Widow	6 (15.4)	4 (10.0)	6 (15.4)	
Divorcee	1 (2.6)	0 (0.0)	1 (2.6)	
No. of pregnancies *	10 [8; 12]	10 [7; 11]	9 [6; 11]	0.299
No. of abortions *	1 [0; 2]	1 [0; 2]	0 [0; 2]	0.369
Gestational diabetes				0.524
Yes	7 (17.9)	4 (10.0)	7 (17.9)	
No	32 (82.1)	36 (90.0)	32 (82.1)	
BMI (kg/m^2^)	29.9 ± 5.5	30.3 ± 6.3	27.6 ± 4.4	0.063
Waist circumference (cm)	99.6 ± 11.1	99.2 ± 12.1	93.7 ± 9.5	0.034
Waist/hip ratio *	1.0 [1.0; 1.1]	1.0 [0.9; 1.1]	1.0 [0.9; 1.1]	0.362
Fat percentage *	45.4 [39.0; 51.0]	47.1 [41.6; 50.6]	43.0 [37.9; 46.6]	0.064
Muscle percentage *	22.1 [20.5; 24.4]	22.2 [20.1; 24.0]	23.0 [21.1; 25.0]	0.233
Visceral fat *	11.0 [8.0; 12.0]	10.0 [9.0; 11.0]	9.0 [8.0; 10.0]	0.008
Biochemistry				
Total cholesterol (mmol/L)	6.2 ± 1.1	6.1 ± 1.1	6.5 ± 1.1	0.394
Triglycerides (mmol/L) *	1.2 [1.0; 1.6]	1.3 [0.9; 1.7]	1.2 [1.0; 1.7]	0.531
LDL (mmol/L)	4.2 ± 1.0	4.1 ± 1.1	4.3 ± 0.9	0.702
HDL (mmol/L) *	1.4 [1.2; 1.7]	1.3 [1.1; 1.5]	1.5 [1.2; 1.8]	0.574
VLDL (mmol/L) *	0.5 [0.5; 0.7]	0.6 [0.4; 0.8]	0.6 [0.5; 0.8]	0.545
Glucose (mmol/L) *	6.0 [5.7; 6.8]	6.2 [5.5; 6.5]	6.0 [5.6; 6.4]	0.862
Apo A1 (g/L) *	1.7 [1.6; 1.9]	1.6 [1.5; 1.9]	1.7 [1.5; 1.9]	0.620
Apo B (g/L) *	1.2 [1.0; 1.4]	1.3 [1.1; 1.4]	1.3 [1.1; 1.3]	0.930
Homocysteine (μmol/L) *	10.2 [8.7; 12.6]	10.2 [8.6; 11.3]	8.4 [7.6; 9.9]	0.003
HbA1c (%) *	5.8 [5.4; 6.1]	5.7 [5.4; 6.0]	5.7 [5.3; 5.9]	0.521
Insulin resistance (HOMA-IR) *	2.4 [1.8; 4.3]	2.4 [1.3; 3.9]	2.3 [1.5; 3.2]	0.412
Hormone levels				
FSH (mIU/mL) *	71.0 [55.5; 91.5]	62.8 [46.0; 83.4]	71.4 [48.3; 87.8]	0.433
Estradiol (pg/mL) *	6.2 [5.0; 13.6]	5.0 [5.0; 7.1]	6.3 [5.0; 11.2]	0.396
Progesterone (ng/mL) *	0.1 [0.1; 0.2]	0.2 [0.1; 0.3]	0.2 [0.1; 0.4]	0.455
Testosterone (ng/mL) *	0.18 [0.06; 0.26]	0.15 [0.10; 0.25]	0.18 [0.12; 0.29]	0.389
LH/FSH *	0.5 [0.4; 0.7]	0.6 [0.4; 1.2]	0.5 [0.1; 0.9]	0.379

Data are presented as mean ± standard deviation (SD) or proportions *n* with corresponding percentages (%). * Skewed variables are presented as median [interquartile range]. ^†^ *p*-values were two-tailed and calculated using one-way ANOVA or Kruskal–Wallis tests, as appropriate. *p*-values < 0.05 were considered statistically significant. Abbreviations: BMI, body mass index; LDL, low-density lipoprotein; HDL, high-density lipoprotein; VLDL, very-low-density lipoprotein; HbA1c, hemoglobin A1c; FSH, follicular stimulating hormone; LH, luteinizing hormone.

**Table 3:** Identification of independently associated variables with serum free thiol levels in reproductive women.

Variables	Univariable Analysis		Multivariable Analysis	
	St. Beta	*p*-Value ^†^	St. Beta	*p*-Value ^†^
Age (years)	0.402	**<0.001**	0.356	**0.002**
Obesity markers				
BMI (kg/m^2^)	0.134	**0.046**	−0.124	0.127
Waist circumference (cm)	0.290	**<0.001**	0.040	0.643
Waist/hip ratio	0.305	**<0.001**	0.143	0.077
Fat percentage	0.102	0.128		
Visceral fat	0.227	**0.001**	−0.132	0.185
Factors associated with CV-risk				
Glucose	−0.021	0.764		
LDL (mmol/L)	0.149	**0.030**	−0.026	0.974
HDL (mmol/L)	−0.084	0.224		
Triglycerides (mmol/L)	0.165	**0.016**	0.025	0.754
Apo A1 (g/L)	−0.003	0.961		
Homocysteine (μmol/L)	−0.206	**0.005**	−0.194	**0.005**
Estradiol (pg/mL)	0.064	0.358		

^†^*p*-values < 0.05 were considered statistically significant. Significances are indicated in bold. Abbreviations: BMI, body mass index; CV, cardiovascular; LDL, low-density lipoprotein; HDL, high-density lipoprotein; Apo A1, Apolipoprotein A1.

**Table 4:** Identification of independently associated variables with free thiol levels in menopausal women.

Variables	Univariable Analysis		Multivariable Analysis	
	St. Beta	*p*-Value ^†^	St. Beta	*p*-Value ^†^
Age (years)	−0.315	**0.001**	−0.291	**0.002**
Obesity markers				
BMI (kg/m^2^)	−0.136	0.142		
Waist circumference (cm)	−0.157	0.090		
Waist/hip ratio	−0.043	0.645		
Fat percentage	−0.103	0.267		
Visceral fat	−0.217	**0.019**	−0.149	0.101
Factors associated with CV-risk				
Glucose	0.110	0.249		
LDL (mmol/L)	0.081	0.414		
HDL (mmol/L)	−0.048	0.614		
Triglycerides (mmol/L)	0.166	0.091		
Apo A1 (g/L)	−0.068	0.474		
Homocysteine (μmol/L)	−0.291	**0.002**	−0.200	**0.032**
Estradiol (pg/mL)	0.023	0.805		

^†^*p*-values < 0.05 were considered statistically significant. Significances are indicated in bold. Abbreviations: BMI, body mass index; CV, cardiovascular; LDL, low-density lipoprotein; HDL, high-density lipoprotein; Apo A1, Apolipoprotein A1.

## Supplementary Materials

Supplementary materials can be found at https://www.mdpi.com/1422-0067/21/1/314/s1.
