# Supplementary material for: Systemic Oxidative Stress Is Increased in Postmenopausal Women and Independently Associates with Homocysteine Levels
Source: Int J Mol Sci. 2020 Jan 2;21(1):314. doi: 10.3390/ijms21010314 (PMC6982320; doi:10.3390/ijms21010314)
Supplement: Supplementary file 1 [file ijms-21-00314-s001.pdf]

Table S1. Cohort characteristics with comparison between reproductive (premenopausal) women and (post)menopausal women.

**Variables**	**Reproductive women**	**Menopausal women**	***P*-value**†
	n = 223	*n* = 118	
Free thiols (μM)	780.3 ± 80.9	762.9 ± 85.3	0.065<br><0.001$^{\times}$
Age (years)*	30 [22;42]	54 [50;59]	< 0.001
<u>Place of living</u><br>Rural<br>Urban	 59 (26.5)<br>164 (73.5)	 5 (4.2)<br>113 (95.8)	< 0.001
<u>Marital status</u><br>Unmarried<br>Married<br>Widow<br>Divorcee	 92 (41.4)<br>125 (56.3)<br>2 (0.9)<br>3 (1.4)	 0 (0.0)<br>100 (84.7)<br>16 (13.6)<br>2 (1.7)	< 0.001
No. of pregnancies*	1 [0;5]	10 [7;12]	< 0.001
No. of abortions*	0 [0;0]	1 [0;2]	< 0.001
<u>Gestational diabetes</u><br>Yes<br>No	 26 (11.7)<br>197 (88.3)	 18 (15.3)<br>100 (84.7)	0.346
BMI (kg/m^2)	26.1 ± 5.9	29.2 ± 5.5	< 0.001
Waist circumference (cm)	83.4 ± 18.2	97.5 ± 11.2	< 0.001
Waist/hip ratio*	0.86 [0.77;0.93]	1.00 [0.92;1.06]	< 0.001
Fat percentage*	40.2 [32.9;47.4]	45.2 [38.5;49.5]	< 0.001
Muscle percentage*	24.2 [22.4;26.4]	22.3 [20.6;24.5]	< 0.001
Visceral fat*	6.0 [4.0;8.0]	10.0 [8.0;11.0]	< 0.001
**Biochemistry**			
Total cholesterol (mmol/L)	4.8 ± 1.1	6.3 ± 1.1	< 0.001
Triglycerides (mmol/L)*	0.8 [0.7;1.2]	1.2 [1.0;1.7]	< 0.001
LDL (mmol/L)	3.0 ± 1.0	4.2 ± 1.0	< 0.001
HDL (mmol/L)*	1.4 [1.2;1.6]	1.4 [1.2;1.7]	0.675
VLDL (mmol/L)*	0.4 [0.3;0.5]	0.6 [0.5;0.8]	< 0.001
Glucose (mmol/L)*	5.6 [5.2;5.9]	6.1 [5.6;6.5]	< 0.001
Apo A1 (g/L)*	1.6 [1.4;1.7]	1.7 [1.5;1.9]	< 0.001
Apo B (g/L)*	0.9 [0.7;1.0]	1.2 [1.1;1.4]	< 0.001
Homocysteine (μmol/L)*	8.8 [7.8;10.2]	9.5 [8.0;11.3]	0.014

HbA1c (%)*	5.4 [5.1;5.6]	5.7 [5.4;6.0]	< 0.001
Insulin resistance (HOMA-IR)*	2.3 [1.6;3.3]	2.4 [1.6;3.8]	0.458
**Hormone levels**			
FSH (mIU/mL)*	6.5 [5.5;8.1]	67.6 [47.6;86.7]	< 0.001
Estradiol (pg/mL)*	37.2 [28.1;47.7]	5.0 [5.0;11.1]	< 0.001
Progesterone (ng/mL)*	0.5 [0.3;0.7]	0.2 [0.1;0.3]	< 0.001
Testosterone (ng/mL)*	0.23 [0.15;0.33]	0.16 [0.11;0.26]	< 0.001
LH/FSH*	0.9 [0.7;1.2]	0.6 [0.3;0.9]	< 0.001

Data are presented as mean ± standard deviation (SD) or proportions n with corresponding percentages (%). *Skewed variables are presented as median [interquartile range]. †*P*-values were two-tailed and calculated using independent sample t-tests or Mann-Whitney U-tests, as appropriate. *P*-values < 0.05 were considered statistically significant. ˣDifference after correction for age, *P*-value derived from linear regression analysis. Abbreviations: BMI, body mass index; LDL, low-density lipoprotein; HDL, high-density lipoprotein; VLDL, very-low-density lipoprotein; HbA1c, hemoglobin A1c; FSH, follicular stimulating hormone; LH, luteinizing hormone.
